# Microbe cultivation guidelines to optimize rhamnolipid applications

**DOI:** 10.1038/s41598-024-59021-7

**Published:** 2024-04-10

**Authors:** Ilona E. Kłosowska-Chomiczewska, Adam Macierzanka, Karol Parchem, Pamela Miłosz, Sonia Bladowska, Iga Płaczkowska, Weronika Hewelt-Belka, Christian Jungnickel

**Affiliations:** 1 Department of Colloid and Lipid Science, Faculty of Chemistry, Gdańsk University of Technology, 11/12 G. Narutowicza St., 80-233 Gdańsk, Poland; 2 Department of Chemistry, Technology and Biotechnology of Food, Faculty of Chemistry, Gdańsk University of Technology, 11/12 G. Narutowicza St., 80-233 Gdańsk, Poland; 3 Department of Analytical Chemistry, Faculty of Chemistry, Gdańsk University of Technology, 11/12 G. Narutowicza St., 80-233 Gdańsk, Poland

**Keywords:** Biosurfactant design, Rhamnolipid biosynthesis, Micellar solubilization, Microbial cultivation, QSPR, Computational biology and bioinformatics, Microbiology, Chemistry, Materials science

## Abstract

In the growing landscape of interest in natural surfactants, selecting the appropriate one for specific applications remains challenging. The extensive, yet often unsystematized, knowledge of microbial surfactants, predominantly represented by rhamnolipids (RLs), typically does not translate beyond the conditions presented in scientific publications. This limitation stems from the numerous variables and their interdependencies that characterize microbial surfactant production. We hypothesized that a computational recipe for biosynthesizing RLs with targeted applicational properties could be developed from existing literature and experimental data. We amassed literature data on RL biosynthesis and micellar solubilization and augmented it with our experimental results on the solubilization of triglycerides (TGs), a topic underrepresented in current literature. Utilizing this data, we constructed mathematical models that can predict RL characteristics and solubilization efficiency, represented as *log*P_RL_ = *f*(carbon and nitrogen source, parameters of biosynthesis) and *log*MSR = *f*(solubilizate, rhamnolipid (e.g. *log*P_RL_), parameters of solubilization), respectively. The models, characterized by robust R^2^ values of respectively 0.581–0.997 and 0.804, enabled the ranking of descriptors based on their significance and impact—positive or negative—on the predicted values. These models have been translated into ready-to-use calculators, tools designed to streamline the selection process for identifying a biosurfactant optimally suited for intended applications.

## Introduction

Microbial surfactants, a subgroup of biosurfactants (BSs) primarily represented by rhamnolipids (RLs), are garnering increasing attention for their potential in various applications. They are widely recognized for being skin-friendly^1,2^, non-toxic^3^, and readily biodegradable^3,4^. Consequently, they are frequently studied to gain a deeper understanding of their behaviour in aqueous solutions under different conditions^5^ and for numerous application purposes^6,7^. Despite having been studied since the 1950s, there remain significant gaps in the field of BS science that warrant further investigation. This study addresses two primary knowledge gaps: I. the applicational properties, specifically micellar solubilization, and II. a targeted synthesis approach for BS research, described further in more detail.

The exploration of the first aspect, i.e. micellar solubilization, seems somewhat incomplete given the myriad combinations of solubilizate and BS. A meticulous review of research papers published since 1995 yielded only 28 publications that provided complete or semi-complete numerical descriptions of the solubilization process in the form of a molar solubilization ratio (MSR). The MSR is the metric most commonly used to describe micellar solubilization efficiency across all surfactants^8^. No reports older than 27 years were identified. A brief analysis of the juxtaposed metadata (SI, “MSR dataset”) suggests that RL solubilization capacities predominantly pertain to compounds with environmental applications. For instance, n-alkanes were featured in 9, and aromatics in 15 of the 28 examples. There is a conspicuous absence of research on solubilization relevant to household contexts (e.g., handwashing, dishwashing), as well as the pharmaceutical or cosmetic industries (such as oil-soluble vitamin supplements or cosmetic emulsions). This research gap might be bridged by undertaking experiments with triglycerides (TGs), which are the primary constituents of vegetable oils.

The second issue, i.e. targeted biosynthesis, is no less significant. Much of the BS research seems to adopt a bottom-up approach. In such a method, research groups identify potential applications for a previously synthesized and analyzed BS, tailoring these applications to the known properties of BSs, rather than the reverse^9–12^. In response to that, we previously proposed a top-down approach for BS. In this strategy, we employed the quantitative structure–property relationship (QSPR) to select BSs with the desired properties for specific targeted applications^8,13^. Our research yielded models that can predict the CMC or MSR of RLs best suited for a chosen solute. Analyzing such models helps in identifying the optimal BS for a given application. Targeted synthesis requires, however, an in-depth and accurate understanding of how selected biosynthesis parameters (e.g., carbon source, nitrogen source, C:N ratio, pH, temperature, etc.) influence BS composition. Typically, such determinations are made to maximize yield^14–19^, with only occasional emphasis on how the parameters affect the final product’s composition. For instance, Nitschke et al.^20^ illustrated how the hydrophilicity of a carbon source impacts the composition of the RL mixture produced by *Pseudomonas aeruginosa* LBI. When cultivated on hydrophobic oil wastes, there was a predominance of monorhamnolipid (monoRL). Similarly, Nicolo et al.^21^ found that *Pseudomonas aeruginosa* PAL05 primarily produces monoRL when grown on substrates providing long-chain fatty acids, such as *Brassica carinata* oil or myristic acid, and dirhamnolipid (diRL) when using glycerol or glucose as the carbon source. Santos et al.^15^ demonstrated that cultivating *Pseudomonas aeruginosa* with different combinations of nitrogen and carbon sources could vary the monoRL and diRL contents in the final product, ranging between 14–67% and 33–85%, respectively. In another study, Santos et al.^22^ revealed that pH not only affects the efficiency of RL production by the *Pseudomonas aeruginosa* PA1 strain but also the mono- to diRL ratio in the final product. Conversely, Costa et al.^23^ and de Santana-Filho et al.^24^ established that the RL congener distribution evolves over the cultivation period, respectively for *Burkholderia glumae* AU6208 and *Pseudomonas aeruginosa* UFPEDA. Both the efficiency of biosynthesis and the congener diversion are influenced by the sulphur source, as Ismail et al. ^25^ found in RL production by *Pseudomonas aeruginosa* AK6U. While these studies offer invaluable insights, they are relatively limited and often restricted to specific microbial species.

A comprehensive meta-analysis, systematic organization, and statistical evaluation of broader RL production data could simplify the selection of biosynthesis conditions. Moreover, integrating such meta-analysis data with application-specific data could potentially serve as a tool for producing surfactants with tailored properties. While there are examples of applying chemometric tools in RL research, they mainly focus on optimizing BS production efficiency^26–28^, aggregation behavior^29^, solubilization and biodegradation of PAHs^30^, treatment of oily sludges^31,32^, coal recovery^33^, and selected adsorption characteristics^34^. To our knowledge, attempts of developing guidelines for the biosynthesis of BSs with specified and targeted properties have not been reported in the scientific literature so far.

Considering all the aforementioned points and aiming to provide a more comprehensive view of BS science, we hypothesized that by generating new experimental data on solubilization of TGs, integrating it with existing literature data on solubilization and biosynthesis, and employing selected chemometric tools, it is possible to formulate a computational blueprint for the biosynthesis of RLs with specific application-oriented properties. Therefore, we aimed at delivering mathematical models to predict (i) the composition of the RL biosynthesized at fixed conditions, and (ii) the efficiency of the obtained RL as a solubilizing agent. We have then transformed the models into ready-to-use calculators for facilitating the biosynthesis of RLs with customized properties.

## Materials and methods

### Materials

The RL preparation R90 was purchased from Agae Technologies (USA). It is a powdered BS containing 90% of RLs with monoRL to diRL ratio 3:2 (w/w). The R90 was used as a source of monoRLs and diRLs. TB (97%, Sigma-Aldrich, W222305) and TO (≥ 99%, Sigma-Aldrich, T7140) were used as solubilizates in solubilization experiments.

Chloroform, methanol, and isopropanol (all ≥ 98.5%) for column chromatography were purchased from POCh (Gliwice, Poland). Gradient grade isopropanol (1.01040), methanol (1.06007), hexane (1.03701) and acetonitrile (1.00030) for HPLC analysis were purchased from MerckKGAA (Darmstadt, Germany). Ammonium formate (98%), sodium azide, sodium hydroxide and Hydrochloric acid were obtained from Chempur (Krupski Młyn, Poland).

Biosurfactant stock solution was prepared in redistilled water (Hydrolab, Straszyn, Poland) with 0.02% (w/w) NaN_3_. Biosurfactant solutions were prepared by serious dilution of the stock solution. Sodium hydroxide and hydrochloric acid solutions were used for pH adjustment.

### Separation of RLs

We used the purchased R90 preparation as a source of RLs, and separated mono- and diRLs with preparative column chromatography. Silicagel (15-40 μm, Merck 60) was activated (105 °C, 3 h) and suspended in chloroform. Glass column (50  cm length, Φ 2 °cm) with a sinter was filled with the silica gel suspension. Subsequently, 2.5 g of BS was dissolved in chloroform and loaded into the column. The elution of RLs was performed according to Sim et al.^35^. Briefly, neutral lipids were eluted with ca*.* 500 mL of chloroform, monoRLs were eluted with ca. 1000 mL of chloroform/methanol (50:3 v/v) followed by 200 mL of chloroform/methanol (50:5 v/v), and diRLs were eluted with ca. 300 mL of chloroform/methanol (50:50 v/v). Eluate fractions were collected in glass vials (10 mL each) at a flow rate 0.5 mL/min, regulated with a membrane pump (APR150, Aqua EL, Suwałki, Poland).

The composition of eluate fractions was analysed using TLC and HPLC Q-TOF methods, and fractions containing either monoRLs or diRLs were combined. The solvents were evaporated using a rotary evaporator (55 °C, 30 min), and the RL congeners obtained that way were further used for solubilization experiments.

### RL separation efficiency assessment

The column chromatography eluate fractions were assessed with thin layer chromatography (TLC) and HPLC coupled to hybrid quadrupole-time of flight analyzer (Q-TOF).

For TLC, eluate fractions of 5 μL were spotted on silica gel plates (TLC 60 F_254_, Merck, Poland). The chromatograms were developed in the ascending mode in a cylindrical developing chamber containing chloroform/methanol/water (65:15:2 v/v/v) under saturated conditions^35^. The spots were visualized by spraying the TLC plate with 10% phosphomolybdic acid in methanol and heated to 100 °C on the heating plate.

The MS analyses were performed using Agilent 1290 LC system equipped with a binary pump, online degasser, autosampler, and a thermostated column compartment, coupled to a 6540 Q-TOF–MS with a dual electrospray ionization (ESI) source (Agilent Technologies, Santa Clara, CA, USA). The extracts were injected directly to the ion source. The mobile phase consisted of 50% 10 mM ammonium formate/methanol (10:90 v/v) (component A) and 50% hexane/isopropanol/5 mM ammonium formate (20:79:1 v/v/v) (component B). The mobile phase flow rate was set to 0.5 mL/min. The injection volume was 2 μL. The ESI source was operated in the positive ion mode with ion spray voltage 120 V. Data were collected using SCAN acquisition mode in a range from 100 to 2200 m/z in high-resolution mode (4 GHz). MS analysis parameters included a capillary voltage of 3500 V, fragmentation voltage of 120 V, nebulizing gas at 35 psi, and a drying gas temperature of 300 °C. Mono- and diRLs were monitored by 522.3636 and 668.4215 m/z ([M^+^NH_4_]^+^ adducts), respectively^36^.

### Solubilization experiments

RL solutions of different concentrations were prepared by a serial dilution of a stock solution. Solubilization was carried out in glass vials (10 mL) with an aperture at the bottom, sealed with silicone septa. Biosurfactant solution (2.5 mL) was transfered into the vial and oil phase (i.e., TB or TO) was added in excess (0.25 mL). Vials were closed with a cap sealed with Teflon septa and mixed thoroughly (1000 rpm, 24 h, 25 °C) using a horizontal shaker (IKA VIBRAX VXR Basic, Sigma-Aldrich) in order to allow for incorporation of oil phase into BS micelles. Subsequently, the excess oil phase was separated by centrifugation at 5000 rpm for 15 min (MPW-350 Med. Instruments, Poland). Immediately after the centrifugation, 1 mL of clear bottom phase was collected with a syringe through silicone septa and stored at 4 °C prior to further analysis. All the solubilization experiments were performed in triplicate.

### Solubilization efficiency assessment

The concentration of TB or TO solubilized in RL micelles was analyzed by reverse phase (RP) UHPLC using Dionex UltiMate 3000 equipped with Corona Veo RS and UV–VIS/DAD detectors, kindly provided by Polygen (Gliwice, Poland). The MSR values of solubilizates were assessed from the slope of the solubility curves above the CMC of BS ^37,38^.

#### Solubilization of TB

Zorbax Bonus RP column (150 × 1.8 mm, 5 µm, Agilent Technologies, USA) was used for the separation of micellar phase components. The mobile phase used for the determination of TB consisted of 5 mM ammonium formate (component A) and acetonitrile (component B). The following gradient elution programme was used: a linear increase of B from 5 to 80% within 4 min, followed by 80% B maintained for 9.5 min. Then, the column was equilibrated for 3 min with 5% B. The flow rate of the mobile phase was 800 µL/min, and the injection volume was 10 µL. The column temperature was maintained at 40 °C throughout the separation process.

#### Solubilization of TO

Poroshell 120 EC-C8 column (100 × 2.1 mm, 2.7 µm, Agilent Technologies, USA) was used for the separation of micellar phase components. The mobile phase used for the determination of TO consisted of water (component A), acetonitrile (component B), and isopropanol (component C). The gradient elution programme was as follows: a linear increase of B from 5 to 95% within 5 min, followed by a simultaneous linear decrease of B to 0%, and an increase of C from 0 to 95% within 5 min. Next, 0% B and 95% C were maintained for 5 min. Subsequently, the column was equilibrated with 5% B and 0% C for 5 min. The flow rate of the mobile phase was 1 mL/min, and the injection volume was 5 µL. The column temperature was maintained at 40 °C throughout the separation process.

### CMC determination

The pendant drop technique was used to assess the CMC of RL solutions (DSA 10, Krȕss, Germany). The tensiometer was calibrated each time before using a RL solution by measuring the surface tension of redistilled water. The solutions used for the assessment of CMC were prepared by serious dilutions of the RL stock solutions that were also used for the solubilization studies. Redistilled water was used to dilute a stock solution to the concentrations required. The equilibrium surface tension measurements were performed at 25 °C. All the measurements were run at least in triplicate. The isotherms of surface tension vs the logarithm of BS concentration were plotted and the CMC was determined from the intersection of two lines, i.e. before and after reaching the plateau of surface tension. The CMC data were further used for the purpose of modeling the efficiency of solubilization in RL solutions, where CMC served as one of the descriptors of the BS.

### Literature data collection for databases

Google Scholar was used for literature data collection as it provides high coverage of published research data^39^. *Rhamnolipid, rhamnolipid production, relative abundance*, *rhamnolipid solubilization, rhamnolipid MSR*, *rhamnolipid WSR,* and *rhamnolipid molar solubilization ratio* were used as keywords. The years 2009–2022 were covered in the search. Only two types of original papers were considered, i.e. publications providing full (continuous) data, and those providing data discontinuous only to the extent, where simple, literature-based assumptions could make them more or fully continuous. Publications giving precise BS composition (i.e. shares of RL homologs) and/or microbiological feed source composition were considered fully continuous and were used without additional calculational interventions. Publications providing discontinuous but still valuable information were supported with some simple assumptions. For example, when authors stated that BS contained 25.65% of monoRL and 74.35% of diRL^22^, without specifying homologs, an average weighted RL composition was calculated for Rha-C10-C10 (one rhamnose and two residues of 3-hydroxydecanoic acid groups) and Rha-Rha-C10-C10 (two rhamnose moieties and two residues of 3-hydroxydecanoic acid groups) as the most abundant mono- and di-RL homologs^40,41^. In turn, when mono- and diRL congeners were given but without pointing monoRL/diRL ratio—like, for example, in the work by Thio et al.^42^—an average 35/65 ratio was assumed as it has been suggested in other studies on RLs^43–45^. Some further assumptions regarding, for example, the use of mixed carbon or nitrogen sources are presented in the Supplementary Information (SI, “RL production dataset”). As a result, data from 75 original publications^21–25,41,42,45–112^ were used for creating the RL production database, resulting in 213 unique data points (SI, “RL production dataset”). For the purpose of creating the MSR database, data from 27 original publications were used^8,13,56,113–135^ (57 literature data points, some of which were used in our previous report^8^) as well as additional original experimental data created for this study (17 data points), resulting in a total number of 74 data points (SI, “MSR dataset”). For modeling purposes, the process of BS synthesis was described by the following descriptors: carbon concentration, the molecular volume of carbon source (MV_C_*)*, the logarithm of octanol–water partition coefficient (*log*P), the concentration of nitrogen, carbon to nitrogen ratio (C:N, w/w), pH of the bacterial medium, incubation temperature and time, shaking speed (V, rpm). RLs were characterized by the logarithm of octanol–water partition coefficient *log*P_RL_, CMC, and Impurity scale (0–5)^8,13^, whereas the solubilization process was characterised by the molecular volume and the logarithm of octanol–water partition coefficient of solubilizate (MV_SOL_ and *log*P_SOL_, respectively), pH, temperature (T), and the efficiency of the process expressed as MSR. Structural descriptors of carbon source and BS (i.e*.* MV and *log*P) were calculated as weighted average for the components/congeners using Molinspiration (Molinspiration Cheminformatics, Slovak Republic)^136^.

When analysing the overall data, 9.6% of the data for the RL production (most commonly pH, followed by shaking speed and time of cultivation), and 1.99% of the data points for the MSR determination (most commonly pH, and temperature) contained missing data. Since the missing data was less than 10% it was decided to use Multivariate Imputation by Chained Equations (MICE) to estimate the missing data. The methodology has been described in detail in Łozińska et al.^137^.

### Computational modelling

An evolutionary algorithm (EA) was used to build the QSPR model by fitting a function to the provided data. For this purpose, GeneXproTools software (v5.0.3926, GepSotf, Portugal) was used. Each dataset was split randomly 80:20 into a training and a validation set, respectively. Three repetitions of each random split were conducted and calculated separately. Each calculation was repeated ten times. Root mean squared error (RMSE) was used as the fitting function. Both the *log*P_RL_ and MSR were calculated. For calculations of *log*P_RL_, the following descriptors were used—(i) bacterial feedstock characteristics: concentration of carbon mg/L, *log*P of carbon source, the concentration of nitrogen (mg/L), C:N ratio, and (ii) culture conditions: pH, temperature, shaking speed (rpm), and time of cultivation. For MSR prediction, the following were used: impurity scale (as described previously^8,13^), *log*P of the BS, *log*P of the solubilizate, the molecular volume of the solubilizate (nm^3^), and CMC of the BS, as well as the temperature and pH of the solubilization process.

### Statistical analysis

The analysis of the importance of each considered descriptor was performed with GeneXproTools software (v5.0.3926, GepSotf, Portugal). Any positive or negative impact of a descriptor on the predicted value was assessed as described previously^12^. Positive contribution (%) was assigned to data points where ∂CMC/∂x > 0, and negative contribution (%) where ∂CMC/∂x < 0.

## Results and discussion

### Computational modelling of RL production database

**Figure 1:**
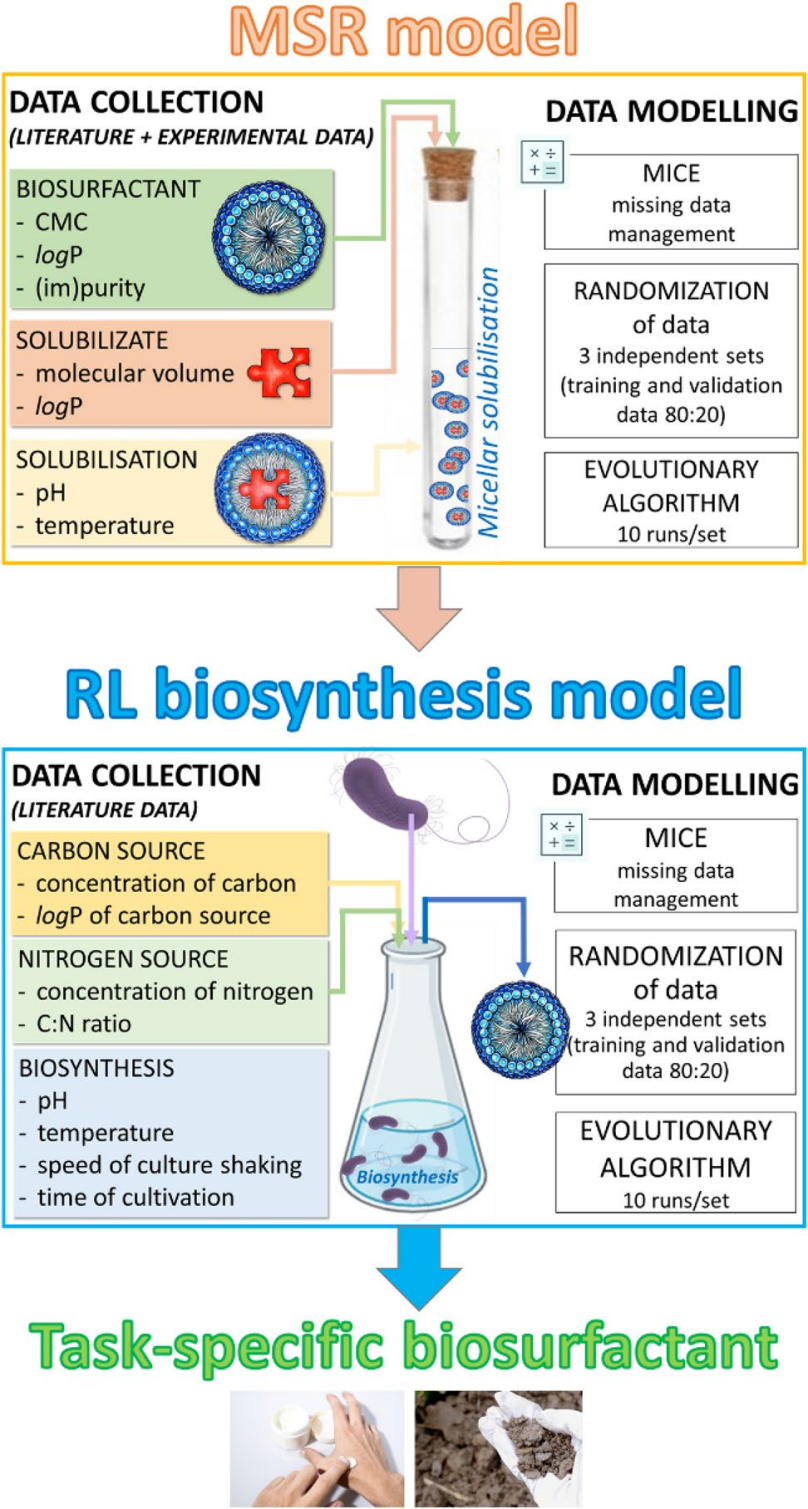
Workflow diagram for creation of RL biosynthesis and MSR models. The diagram also summarizes the way in which the models were used to define a recipe for a task-specific BS.

**Figure 2:**
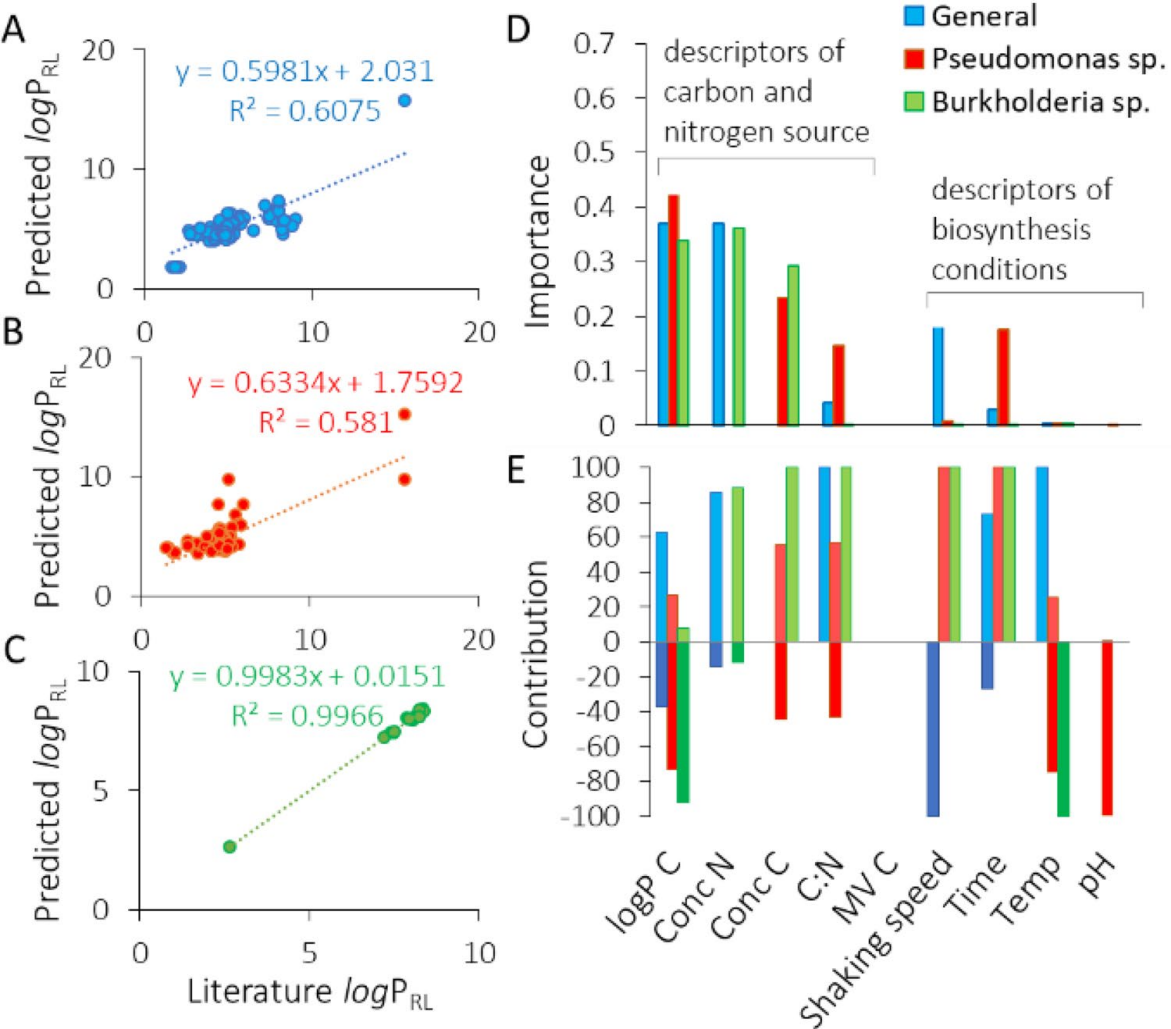
The estimated predictive power of the RL biosynthesis models: the general model for collated data (**A**) and separate models for Pseudomonas (**B**) or Burkholderia species (**C**). The importance of model variables (**D**) with respect to their positive or negative contribution to the predicted value (**E**). Model variables include descriptors of carbon and nitrogen sources as well as descriptors of environmental conditions of biosynthesis: *logPRL* and *logPC* logarithm of octanol–water partition coefficient for RL or carbon source, respectively, *Conc N* and *Conc C* concentration of nitrogen or carbon source, respectively, *C:N* carbon to nitrogen ratio, *MVC* the molecular volume of carbon source, *Temp* temperature.

For the purpose of creating task-specific BS, we have first created a general model to describe the influence of carbon and nitrogen sources and the conditions of biosynthesis on the type of the RL product characterized numerically by its *log*P. This is referred to as the ‘RL biosynthesis model’ throughout the manuscript (Fig. 1). The general RL biosynthesis model was based on all collected data. The model was characterized by a good coefficient of determination R^2^ of 0.608 (Fig. 2A). In the second approach, we aimed to analyze the effect of RL producers separately. This was because the cultivation conditions, the metabolic pathways, etc., may vary substantially between different types of RL-producing microbes, which can secrete different RL products. Therefore, we created separate models for the most represented groups of producers, i.e., *Pseudomonas* and *Burkholderia* species (Fig. 2B,C). The coefficient of determination obtained for the RL biosynthesis model for *Pseudomonas* species was slightly lower (R^2^ 0.581) than for the general RL biosynthesis model, indicating that *Pseudomonas* strains were too varied to be characterized by the descriptors widely available in literature data. On the other hand, a very satisfying R^2^ of 0.997 was obtained for the RL biosynthesis model for *Burkholderia* species. This may indicate that the descriptors that were chosen for modeling RL production database describe differences between *Bulkholderia* strains well, or that the *Burkholderia* strains were less varied than the *Pseudomonas* strains. All three RL biosynthesis models were analyzed towards the importance of model descriptors (Fig. 2D). In general, descriptors of carbon and nitrogen source were the most important in all the models created, with *log*P of carbon (*log*P_C_) scoring the highest values among all the descriptors (importance of 0.37, 0.43 and 0.34 for the general, the *Pseudomonas* and the *Burkholderia* models, respectively). Both, the general and the *Burholderia* models showed high importance of nitrogen concentration (importance of 0.37 and 0.36, respectively). The concentration of carbon was the third most important descriptor, with an importance value of 0.23 for the *Pseudomonas* and 0.29 for the *Burkholderia* model. The C:N ratio was the fourth most important descriptor out of the group of carbon and nitrogen descriptors (importance of 0.04, 0.15 and 0.001 for the general, the *Pseudomonas* and the *Burkholderia* models, respectively) and fifth out of all examined descriptors in the general and the *Pseudomonas* models. In the general model, the C:N ratio was outperformed by the speed of shaking the culture (V, importance of 0.18) and the time of cultivation (importance of 0.17). Development of such models for predicting characteristics of the surface active products of biosynthesis is, to our best knowledge, presented for the first time. However, it is impossible to make a direct comparison of the obtained results with the literature. Most similar models that exist in the field of biosynthesis consider either enzymatic synthesis of cationic surfactant^138^ or enzyme biosynthesis^139^. Masoumi et al*.*^138^ have successfully predicted optimal conditions and enzyme amounts for enzymatic synthesis of triethanolamine based esterquat cationic surfactant. The model was characterized by a high coefficient of determination (R^2^_training set_ = 0.9079, R^2^_test set_ = 0.9315). Such a good fit is probably due to omitting the stage of microbial production, as the enzyme was applied. In turn, Banerjee and Bhattacharyya^139^ have optimized the effect of inducers on protease biosynthesis by *Rhizopus oryzae*. Although the optimization resulted in a 2.5-fold increase in efficiency, the R^2^ = 0.0037 (calculated from the data provided by the authors) of the model is definitely not a satisfying outcome.

An indirect comparison of the RL biosynthesis model with literature data can be based on the analysis of the effect of descriptors on the predicted parameter (*log*P_RL_). When an increase in the numerical value of a descriptor leads to an increase in the value of a predicted parameter, a positive effect is credited to such a descriptor. Conversely, if a decrease in the value of a predicted parameter is observed, a negative effect is credited. And so, a positive effect of the hydrophobicity of carbon source (*log*P_C_) was observed in 63% cases in the general RL biosynthesis model, 27% in the *Pseudomonas* model and only 8% in the *Burkholderia* model (Fig. 2E). The results obtained by Nicolo et al.^21^ also presented positive effect. When they cultivated *Pseudomonas* on low-*log*P-carbon-sources (*log*P_C_ of − 1.6 and − 2.64 for glycerol and glucose, respectively) preferentially low-*log*P-biosurfactant product was produced, i.e. BS dominated by diRL (*log*P_RL_ of 4.69 and 4.80 when growing on glycerol and glucose, respectively). In turn, when grown on high-*log*P-carbon-sources (*log*P_C_ of 6.05 and 10.84 for myristic acid and *brassica carrinata* oil, respectively), high-*log*P-biosurfactant product was obtained, where monoRL homologs were dominating (*log*P_RL_ of 4.88 and 5.15, respectively) (see SI, “RL production dataset”). A negative effect was observed *for Burkholderia glumae*^23^, where cultivation on glycerol (*log*P_C_, − 1.6) resulted in producing BS containing 98–99% diRL (*log*P_RL_ 7.95–8.04), whereas 88–96% of diRL (*log*P_RL_, 7.20–7.52) was obtained when using rapeseed oil (*log*P_C_ 10.72) (see SI, “RL production dataset”).

The effect of the second most important descriptor for the two models, the concentration of nitrogen, was mostly positive (in 85% and 88% cases in the general and the *Burkholderia* models, respectively, Fig. 2E). A vague positive effect was observed by Mata-Sandoval et al.^140^ during the cultivation of *Pseudomonas* UG2 on corn oil and using (NH_4_)_2_SO_4_ as a nitrogen source. The authors found that adding nutrients at once (higher starting concentration of nitrogen) resulted in a significant decrease in the share of diRL in the BS mixture (an increase of *log*P_RL_) compared to adding nitrogen in six fractions at two-day intervals.

The third important descriptor, the concentration of carbon, had a rather positive or even exclusively positive effect on the RL hydrophobicity (55% and 100% cases in the *Pseudomonas* and the *Burkholderia* model, respectively, Fig. 2E). This suggests that increasing the concentration of carbon favors production of monoRL. The C:N ratio revealed mostly or exclusively positive contribution (100, 57 and 100% in the general, the *Pseudomonas* and the *Burkholderia* models, respectively). The descriptors of the biosynthesis conditions were mostly of a statistically insignificant importance to the models, except for the speed of shaking the culture in the general model and the time of cultivation in the *Pseudomonas* model (Fig. 2D). The contribution of the first one was 100% negative, whereas the contribution of the second one—100% positive (Fig. 2E). Nitschke et al.^49^ and de Santana-Filho et al.^24^ have found a negative influence of the time of cultivation on the hydrophobicity of RL produced by *Pseudomonas aeruginosa* LBI and *Pseudomonas aeruginosa* UFPEDA 614, respectively. They have stated that monoRLs are produced first, and with time they are transformed into diRLs. A negative effect of time was also observed for *Burkholderia glumae* AU6217 by Costa et al.^23^ when grown on glycerol. But when grown on rapeseed oil, the effect was positive (SI, “RL Production Dataset).

### Solubilization of triglycerides

Two TG species were chosen as solubilizates for experiments, namely tributyrin (TB) and triolein (TO). The MSR values estimated for the purpose of this study were in the range of 1.1 × 10^−5^ for TO solubilized in diRL solutions to 3 × 10^−3^ for TO solubilized in alkaline monoRL solutions, and were, in general, considerably lower than 90% of the collected literature MSR values (those ranged from 3 × 10^−3^ ^134^ for phenanthrene in RL solution to 7.44^124^ for naphthalene in RL mixture) (SI, “MSR dataset”).

#### MonoRL

**Figure 3:**
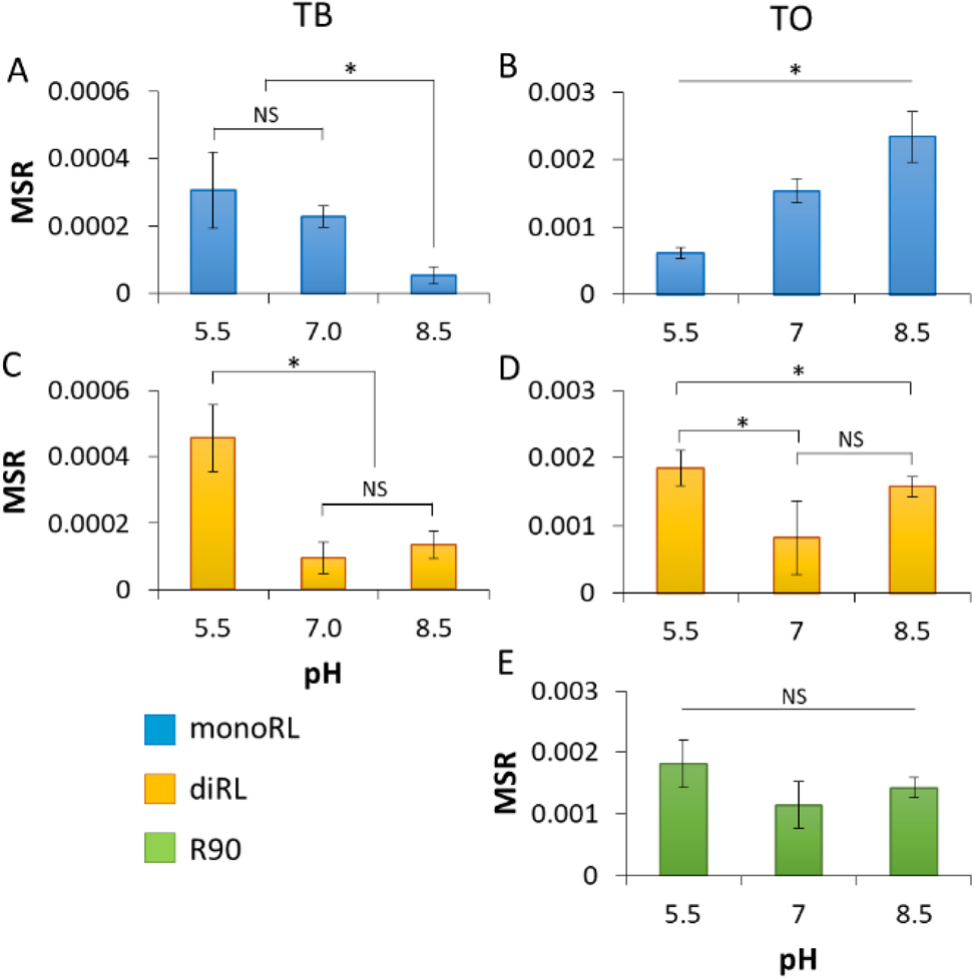
Influence of pH on the efficiency of micellar solubilization, expressed as molar solubilization ratio (MSR) for TB (**A**, **C**) and TO (**B**, **D**, **E**) in solutions of monoRL (**A**, **B**), diRL (**C**, **D**) or their mixture (R90) (**E**). *P < 0.05, by ANOVA; NS, P > 0.05, by ANOVA.

The efficiency of TB solubilization in monoRL solutions decreased with increasing pH, with the average MSR_TB_ of 3.1 × 10^−4^ at pH 5.5 and only 5 × 10^−5^ at pH 8.5 (Fig. 3A). This is in accordance with previous observations of Dahrazma et al.^141^ and Eismin et al.^5^ who stated that when increasing the pH, the structure of RL aggregates change from large to small vesicles and further to micelles, followed by a significant decrease of solubilizing capacity. However, the increase of MSR observed for TO upon increasing pH (Fig. 3B) is in contradiction with that statement. It suggests that the solubilization capacity is not solely a function of the surfactant aggregate type, but also the geometry of the surfactant and solubilizate molecules. Thus, the smaller and more hydrophilic TB (MV 297.46 Å^3^, *log*P 3.27) probably is located in the core and palisade layer of aggregates, similarly as observed by Lin et al*.*^142^ for solubilization of TB in diheptanoylphosphatidylcholine micelles^142^. In turn, hydrophobic and much bigger TO (*log*P 10.78, MV 984.58 Å^3^) locates rather alongside monoRL molecules. We speculate that the double bond-dependent flexibility of the TO hydrophobic tails allows for fitting easily between RL molecules—an arrangement similar to how TO molecules locate themselves in mixed bilayers with phosphatidylcholine (PC)^143^. A previous ^13^C NMR study showed that TO is located between PC molecules with carbonyl groups exposed to bulk solution. A similar molecular structure of PC and RL (i.e. both are double-tailed surfactants), as well as similar values of critical packing parameters (CPP of 0.62 for monoRL, 0.73 for diRL^144,145^, and 0.60–0.61 for PC^146^) might suggest that TO located in the same manner in the RL systems examined here. Besides, the TO hydrocarbon tails are much longer (C18) than the hydrocarbon tails of TB (C4). Therefore, the entropic penalty to incorporate TO into a micelle is bigger. The hydrocarbon tails of TO are longer than those of RLs too (C7 hydrophobic moiety in β-hydroxydecanoic acid). Thus, when incorporated into a micelle, the TO molecules must fit between the surfactant molecules, therefore, pushing away one RL molecule from another and artificially extending the tail of the surfactant by several carbons^141^. Such extended tails form a new hydrophobic core of the micelle and consequently, the aggregate expands/swells. Distant RL molecules are more resistant to pH-induced micelle-rearrangement. Thus, in contrast to the solubilization of TB (Fig. 3A), we did not observe a decrease in the TO solubilization efficiency upon increasing the pH (Fig. 3B). Contrary to TO, TB does not have the ability to swell micelles^142^.

As reported previously in the scientific literature, the *p*K_a_ values for RLs are in the range of 4.28 to 5.60^147,148^. Therefore, the higher the pH, the more dissociated RL is and the bigger the electrostatic repulsion between slightly acidic rhamnose head groups. Such repulsion virtually increases the size of the surfactant head and must rearrange the geometry of the micelle, where the created virtual spaces can be easily filled with TO molecules. This might explain the increase of MSRTO that we observed when increasing the pH (Fig. 3B). Opposite tendency has been observed by Luning Prak et al*.*^149^, where the efficiency of solubilization was decreasing with an increase of the hydrocarbon chain length of the solubilizate. However, the solubilization in the aforementioned research was carried out for *n*-alkanes. Such hydrophobic molecules are incorporated in the hydrophobic core of the micelle, not in the palisade layer^150^.

#### DiRL

Contrary to monoRL, the effect of pH on diRL solutions was less clear (Fig. 3 C,D). The highest average MSR values for both TB and TO were observed at a pH of 5.5 (4.6 × 10^−4^ and 1.8 × 10^−3^, respectively). At this pH, RLs are either not dissociated or only slightly dissociated, as indicated by their low pK_a_ values (4.28 to 5.60^147,148^). Due to their rather nonionic behavior under these conditions, it is primarily the geometry that influences the packing of solubilizates into micelles. With two rhamnose moieties, diRL aggregates provide additional space in the palisade region. This allows for a greater quantity of both solubilizates to be incorporated compared to monoRL at pH 5.5 (MSR_TB_ = 3.1 × 10^−4^ and MSR_TO_ = 6 × 10^−4^, Fig. 3A,B). This observation aligns with the findings of Luning Prak et al.^149^, which showed an increase in solubilization efficiency with the increase in polar heads of nonionic surfactants. Conversely, Zhang et al.^122^ reported an opposite phenomenon, where the solubilization efficiency for phenanthrene was higher in monoRL than in diRL solutions, with MSRs of 5.7 × 10^−2^ and 2.1 × 10^−2^, respectively. However, this could be attributed to the greater hydrophobicity of phenanthrene compared to the TG species examined in our research, resulting in a different solubilizate location within the micelle.

With an increase in pH to 7, we observed a significant decrease in average solubilization efficiency in diRL solutions, whereas an increase in pH to 8.5 resulted in enhanced average efficiency (Fig. 3C,D). This phenomenon may be attributed to the increasing repulsion forces between the large diRL headgroups, which consequently expands the palisade region space. In an alkaline environment, the small TB molecule fits into micelles even more effectively than in monoRL solutions, yielding a MSR value of 1.4 × 10^−4^ (Fig. 3C) as compared to 5 × 10^−5^ in monoRL solutions (Fig. 3A). However, this is not the case for TO, because it requires space not only in the palisade layer but also along the surfactant molecules. The repulsion, however, may not be sufficient for TO, as diRL is less ionic than monoRL even at a pH of 9, a behavior attributed to different intramolecular interactions and conformational changes^54^. This leads us to speculate that the solubilization capacity in diRL solutions is influenced more by geometric factors (overall size) and the resulting hydrophilicity (ratio of moieties) of BS molecules, rather than by electrostatic interactions. Therefore, the palisade layer of the micelle, under conditions favorable for TB and TO, shifts towards the core of the micelle due to the presence of a second rhamnose moiety. This shift results in a more pronounced decrease in TB solubilization, as TO locates along the BS molecules and causes micelle swelling, a phenomenon previously described for monoRLs (3.2.1. MonoRL). Consequently, the MSR values for solubilization of TO in diRL solutions were again higher (Fig. 3D) than those obtained for TB (Fig. 3C).

#### R90

The influence of pH on MSR values for TO solubilization in R90 (Fig. 3E), being the mixture of mono- and diRL, was more akin to that observed in diRL (Fig. 3D) than in monoRL solutions. Namely, the highest average MSR value was observed at pH 5.5, followed by pH 8.5 and 7. Considering it is monoRL that prevails R90 in mass (mono- and diRL 3:2 (w/w)), we can speculate that mixed micelle attributes are governed rather by bigger but less ionic diRL. Further, the average MSR values for R90 measured at different pH did not differ significantly (P > 0.05, Fig. 3E), again underlying the similarity of R90 to diRL.

#### Summary

The obtained data indicate that the efficiency of solubilization results from both surfactant and solubilizate characteristics. Therefore, it seems reasonable to hypothesize that solubilization efficiency can be statistically predicted by modeling nonlinear dependencies on BS, solubilizate, and environmental descriptors. The experimental data on TGs solubilization were subsequently utilized to augment the database of solubilizates, allowing the model to encompass all potential applications of BS solutions in predicting the MSR. This new experimental data accounts for 23% of the entire dataset of MSR values compiled for this study (SI, “MSR dataset”).

### Modelling solubilizing potential of BS

**Figure 4:**
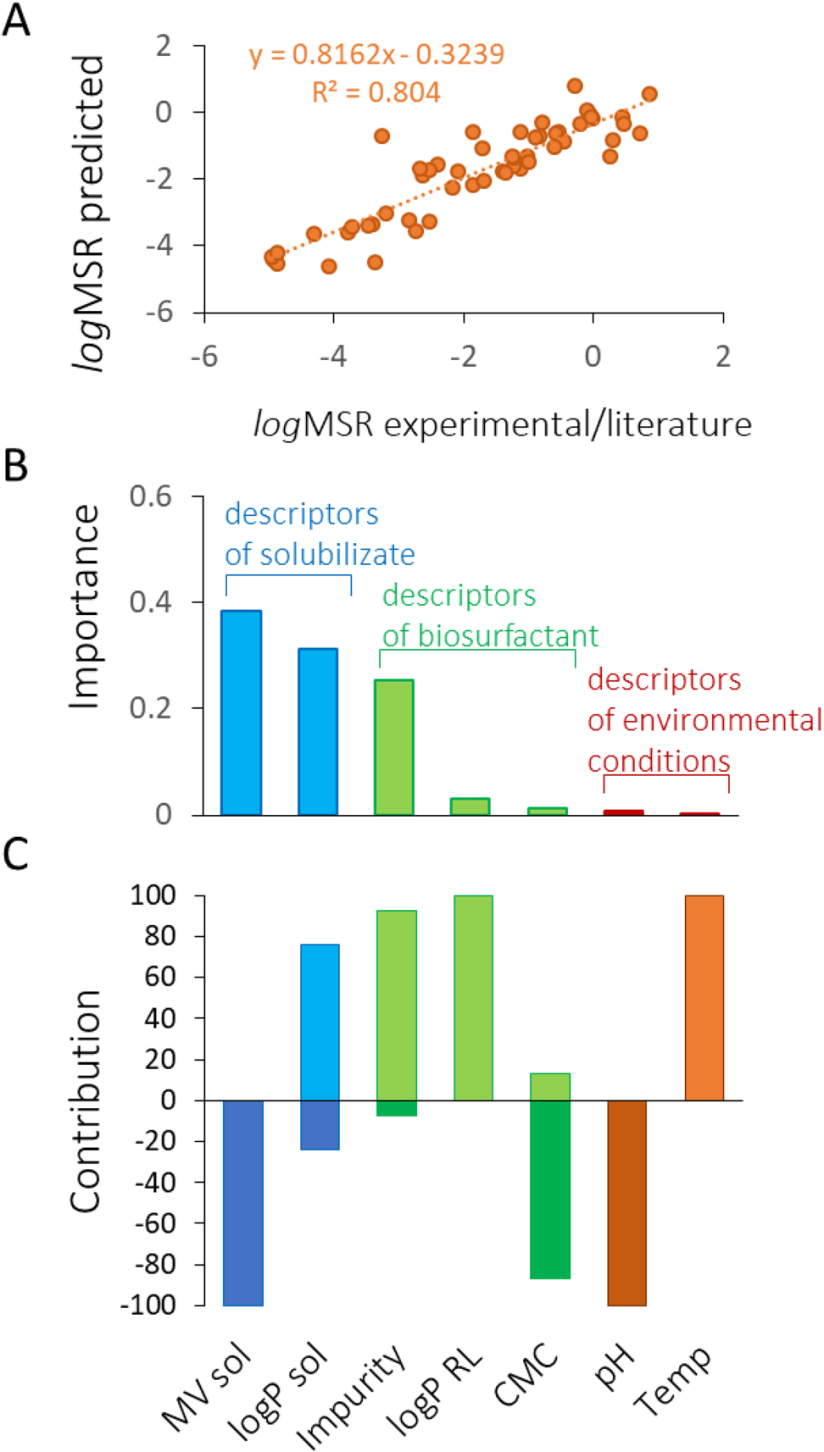
The estimated predictive power of the MSR model for calculating the solubilizing potential of BS, represented by logMSR (**A**), and the importance (**B**) and positive or negative contribution of various model variables (**C**). These variables encompass descriptors of the solubilizate, BS, and solubilization conditions, including MVsol (molecular volume of the solubilizate), *logPSOL* and *logPRL* logarithms of the octanol–water partition coefficients for solubilizate and *RL* respectively, *Impurity* based on a reversed BS purity scale^8,13^, *CMC* critical micelle concentration, and *Temp* temperature.

Having expanded the MSR database with the newly obtained results on TGs solubilization, we developed a model to predict the influence of BS and solubilizate descriptors, as well as process conditions, on the efficiency of micellar solubilization. The MSR model demonstrates a good predictive power, with a coefficient of determination R^2^ of 0.804 (Fig. 4A). Intriguingly, this model, which encompasses a broad spectrum of potential solubilizates, exhibits even greater predictive accuracy than our previous model (R^2^ 0.773)^8^. The latter was developed for a much narrower range of solubilizates and specifically did not include data on TG species solubilization, as such data, to the best of our knowledge, were not previously available in the literature.

The analysis of the descriptors for their importance to the model clearly revealed that solubilizate characteristics are most crucial for efficient solubilization. The molecular volume of solubilizate (MV_SOL_) showed the highest importance with a value of 0.384, followed by *log*P_SOL_ with 0.312 (Fig. 4B). The third most important was the Impurity of BS, at the importance of 0.252. In comparison, the overall group of BS descriptors had lesser importance, with 0.031 and 0.013 for *log*P_RL_ and CMC, respectively. This ranking places BS descriptors after those of the solubilizate. Environmental conditions, such as pH and temperature, were the least important in the MSR model, with respective importances of 0.0083 and 0.0002.

**Figure 5:**
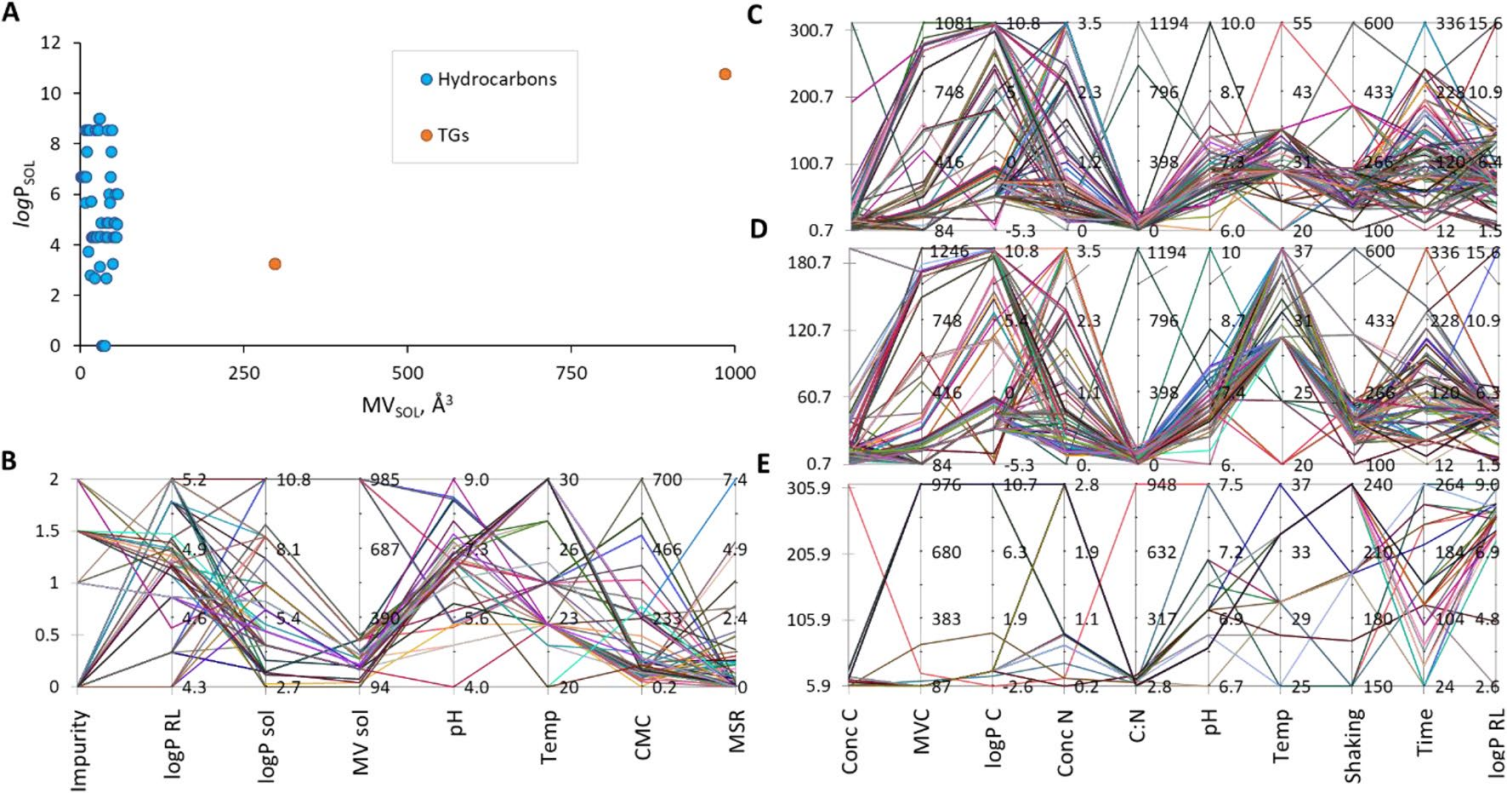
Comparative analysis of solubilizate descriptors for hydrocarbons and TGs, illustrating their respective potential to broaden the applicability domain of the model (**A**). Applicability domains for the MSR model (**B**), RL biosynthesis model (**C**), Pseudomonas model (**D**) and Burkholderia model (**E**) are presented in the form of parallel coordinates. Y-axis of applicability domains represents absolute values of the model descriptors: *Impurity* impurity of biosurfactant, *logPRL,* *logPsol* and *logPC* logarithm of octanol–water partition coefficient for RL, solubilizate or carbon source, *MVsol* and *MVC* molecular volume of solubilizate or carbon source in Å3; pH; *Temp* temperature in °C; *CMC* critical micelle concentration of RL in mg/L; *MSR* molar solubilization ratio, *ConcC* and *ConcN* concentration of carbon or nitrogen in mg/L, *C:N* carbon-to-nitrogen ratio; *Shaking* speed of shaking microbiological culture in rpm; *Time* time of biosynthesis in h. Each colored line represents one observation.

These findings align with our experimental results, where solubilizate characteristics (i.e., geometry and hydrophilicity) significantly influenced micellar solubilization in both mono- and diRL solutions. In contrast, pH showed a clear influence and trend only in the case of monoRL (Fig. 4). Notably, for the bioMSRcalc model^8^, environmental conditions were more influential, followed by BS descriptors and solubilizate descriptors. This variation suggests that including MSR data for the solubilization of TG species is vital not only for the model’s predictive power but also for accurately determining the importance of descriptors. The differences in hydrophobicity (*log*P of 3.27 and 10.78 for TB and TO, respectively) and molecular volume (MV of 297.46 and 984.58 Å^3^ for TB and TO, respectively) of the TG species used in our experiments are substantial. These characteristics could notably expand the applicability domain of the model^151^, as shown in Fig. 5A.

The MV of solubilizate exhibited a completely negative influence on the *log*MSR (100%, Fig. 4C). This is logical, considering that larger molecules are more challenging to incorporate into micelles. In turn, the hydrophobicity of the solubilizate (*log*P_SOL_) showed a predominantly positive contribution (76%), indicating that the more hydrophobic the solubilizate, the higher the MSR. Concerning the impurity of BS, which is another significant descriptor (importance of 0.252, Fig. 4B), it also demonstrated a mainly positive effect (93%, Fig. 4C). This can be explained by the concept of competitive solubilization^152^. When impurities from insufficiently purified BS preparations compete for the same space in the micelle as the solubilizate does, they occupy areas typically reserved for the solubilizate, thus potentially reducing solubilization efficiency. Conversely, if the impurities occupy different locations within the micelle, this can lead to micellar swelling, thereby creating more space for the solubilizate and enhancing solubilization efficiency. According to the MSR model, impurities accompanying RL tend to cause micellar swelling, thereby increasing the efficiency of micellar solubilization.

### Applicability domain of the models

Quantitative-Structure–Property Relationships (QSPR) are recommended by the European Community (e.g., REACH Article 1^153^) to aid in identifying the activity or properties of chemicals. However, these models are not without limitations. Primarily, the availability of experimental data in published works is constrained, and comparing results across different laboratories is challenging due to varying assumptions and methodologies. This heterogeneity leads to fragmented datasets and restricts the selection of model descriptors to those most commonly reported in the literature. Moreover, like all QSPR models, their accuracy is confined within their applicability domain—that is, the range of descriptor values on which the model was trained. Extrapolating results beyond this domain can introduce errors^151^. The applicability domains for the MSR model, the RL biosynthesis model, and the models for *Pseudomonas* and *Burkholderia* are illustrated in Fig. 5B–E. For instance, the MSR model applies to solubilizates with *log*P values between 2.7 and 10.6 and molecular volumes (MV) from 94 to 985 Å^3^ (Fig. 5B). Detailed numeric descriptions of each model’s applicability domain are provided in the supplementary information (SI—Equations, Tab.1).

## Conclusions

In this study, we have combined three novel scientific endeavors. We conducted experimental investigations to describe the micellar solubilization of TG species in BS solutions. Additionally, we compiled databases from both literature and experimental sources concerning RL production during biosynthesis and RL application in the solubilization of various solubilizates. Utilizing these databases, we developed mathematical models that predict I. the composition of biosynthesized RLs based on biosynthesis parameters, and II. the efficiency of micellar solubilization considering the characteristics of RLs, the solubilizate, and the environmental conditions.

Our findings demonstrate that the characteristics of RLs, indicated by *log*P_RL_, can be successfully predicted from the descriptors of carbon and nitrogen sources, and the conditions of microbial species cultivation. Among the three RL production models, the *Burkholderia* sp. model showed the highest coefficient of determination, followed by the general and the *Pseudomonas* sp. models.

In all three models, descriptors of carbon and nitrogen sources emerged as the most significant factors influencing the composition of the produced RLs. Notably, the hydrophobicity of the carbon source (*log*P_C_) was identified as the most crucial parameter across the models. However, the influence of *log*P_C_ on the characteristics of the produced BSs varied between the models.

The efficiency of micellar solubilization in RL solutions exhibited variations across different solubilizates, RL congeners, and pH conditions. We observed a distinct trend of pH influence, specifically in solubilizations using monoRL, which was not observed for diRL. Interestingly, the solubilization efficiency in R90 was more akin to that in diRL, despite monoRL being predominant in its composition.

By integrating our new experimental data with previous research^8^ and a critical review of the latest literature, we have shown that the three most influential descriptors—MV and *log*P of solubilizate, as well as BS impurity—all had substantial effects on solubilization efficiency.

These models offer insights into how to cultivate microorganisms to produce BSs with specific solubilization properties. They can serve as computational guides in selecting the most suitable BS for solubilizing a desired substance in a formulation. For instance, in the context of environmentally friendly removal of contaminants from surfaces, one could first calculate the desired *log*P of a BS, providing required MSR range using the MSR calculator (SI, MSR calculator) along with possible environmental conditions. In the next step, using calculated *log*P_BS_, one could determine cultivation conditions required to produce the requested BS (SI, RL production calculator).

To the best of our knowledge, this approach represents the first comprehensive methodology for developing RL-BS tailored to specific solubilization needs.

### Supplementary Information

Supplementary Information 1.

Supplementary Information 2.

Supplementary Information 3.

Supplementary Information 4.

Supplementary Information 5.

## Data Availability

All data generated or analysed during this study are included in this published article (and its Supplementary Information files).

## Abbreviations

BS: Biosurfactant
CMC: Critical micellar concentration
*log*P: Logarithm of octanol–water partition coefficient
MSR: Molar solubilization ration
MV: Molecular volume
RL: Rhamnolipid
TB: Tributyrin
TG: Triglyceride
TO: Triolein
