# Supplementary material for: Microbe cultivation guidelines to optimize rhamnolipid applications
Source: Sci Rep. 2024 Apr 10;14:8362. doi: 10.1038/s41598-024-59021-7 (PMC11006924; doi:10.1038/s41598-024-59021-7)
Supplement: Supplementary file 1 — Supplementary Information 1. [file 41598_2024_59021_MOESM1_ESM.docx]

Microbe Cultivation Guidelines to Optimize Rhamnolipid Applications

*Ilona E. Kłosowska-Chomiczewska ^a^*, Adam Macierzanka ^a^, Karol Parchem ^b^, Pamela Miłosz ^a^, Sonia Sarach ^a^, Iga Płaczkowska ^a^, Weronika Hewelt-Belka ^c^ and Christian Jungnickel ^a^*

^a^ Department of Colloid and Lipid Science, ^b^ Department of Chemistry, Technology and Biotechnology of Food, ^c^ Department of Analytical Chemistry, Faculty of Chemistry, Gdańsk University of Technology, Narutowicza St. 11/12, 80-233 Gdańsk, Poland

* Corresponding author: ilochomi@pg.edu.pl, tel: +48 58 347 1151 (Ilona E. Kłosowska-Chomiczewska)

ABBREVIATIONS:

BS, biosurfactant

CMC, critical micellar concentration

*log*P, logarithm of octanol-water partition coefficient

MSR, molar solubilization ration

MV, molecular volume

RL, rhamnolipid

TB, tributyrin

TG, triglyceride

TO, triolein

**Simplified equations to calculate *log*MSR in MSR model or *log*PRL in General RL production, *Pseudomonas* or *Burkholderia* models.**

**MSR MODEL:**

*log***MSR** = gep3Rt((-5.98565324869533 × Impurity + *log*P_sol_)^2^) + gep3Rt(*log*P_BS_^3^) - gep3Rt(MV_sol_ - *log*P_sol_ ^2^) + gep3Rt(gep3Rt((*log*P_sol_ × 0.796361920424713 ^CMC / Temp^)^2^)) + Impurity ^1/64^ × gep3Rt( CMC^1/3^ - *log*P_sol_ ^1/2^) - 4.49562508101673 – ((pH/T) *^log^*^Psol / pH^ ) ^3/4^

**GENERAL RL PRODUCTION MODEL:**

***log*P_RL_** = gep5Rt(1/ ( V / 49.2142267718464 - 2.04231706261052))

+ gep3Rt(Exp(gep5Rt(*log*P_C_ + Exp(gep5Rt(C:N + *log*P_C_)))))

+ (ConcN ^2^ / (gep5Rt(1.24487061674623 × Time/Temp)^4^))^1/2^

+ (gep3Rt((gep3Rt(Time/ *log*P_C_))^4^) -1.45700258207625)^1/2^

+ gep3Rt((Exp(Exp(1/( -2.01704920584773 · *log*P_C_ - 1/ConcN ))))^1/2^)

***PSEUDOMONAS* MODEL:**

***log*P_RL_** = (Time × Temp / (*log*P_C_ ^3^- C:N/ConcC))^4^

+ (1 / ((V/ 8.18633453812374)^1/2^ /( V × pH)))^1/2^ / pH

+ gep5Rt((0.46453042630744 × pH /( Temp - C:N - 1.49254419786391))^2^)

+ Exp(( 5.94500650170427 × (Temp / 2.80488167947401 - ConcC - 10.2801284924995) / ( Temp - pH))^5^)

+ Exp(((Time / *log*P_C_+ Time)/ Temp)/(Time - *log*P_C_)^1/2^ )

***BURKHOLDERIA* MODEL:**

***log*P_RL_** = 348.004920604344 × V × ConcN/Temp^4^ + 4.3191306986188

+ 1/(gep5Rt(ConcC × (*log*P_C_ + 2.66806275829075)^1/2^ /((C:N)^1/3^)^5^)

+ gep5Rt(ConcC - gep5Rt(*log*P_C_)/0.165506506650498)

+ gep5Rt(ConcC - *log*P_C_ + gep5Rt(Time – 2 × ConcC ))

+ gep3Rt(*log*P_C_)/(8.26111447795038 × ConcN ^5/2^)

Function gep3Rt(x):

If x < 0.0 then gep3Rt = -((-x)^1/3^), else gep3Rt = x^1/3^

Function gep5Rt(x):

If x < 0.0 then gep5Rt = -((-x)^1/5^), else gep5Rt = x^1/5^

**Tab. 1.** Numerical description of applicability domains for mathematical models: MSR model, general RL production model, *Pseudomonas* and *Burkholderia* models. Minimal, maximal, mean values and standard deviation of model descriptors are given.

|  | **MSR model** | | | | | | | |  |  |  |  |  |  |  |  |
| --- | --- | --- | --- | --- | --- | --- | --- | --- | --- | --- | --- | --- | --- | --- | --- | --- |
| Model descriptor | Minimum | | | Maximum | | Mean | | Std. deviation |  |  |  |  |  |  |  |  |
| Impurity, [0-5] | 0.00 | | | 2.00 | | 0.49 | | 0.73 |  |  |  |  |  |  |  |  |
| *log*P RL | 4.37 | | | 5.21 | | 4.83 | | 0.25 |  |  |  |  |  |  |  |  |
| *log*P of solubilizate | 2.69 | | | 10.78 | | 6.06 | | 2.52 |  |  |  |  |  |  |  |  |
| MV sol, Å^3^ | 94.19 | | | 984.58 | | 309.40 | | 259.21 |  |  |  |  |  |  |  |  |
| pH | 4.00 | | | 9.00 | | 6.95 | | 0.84 |  |  |  |  |  |  |  |  |
| Temperature, °C | 20.0 | | | 30.0 | | 25.1 | | 2.9 |  |  |  |  |  |  |  |  |
| CMC, mg/L | 0.2 | | | 700.0 | | 158.4 | | 163.6 |  |  |  |  |  |  |  |  |
| MSR | 0.00 | | | 7.44 | | 0.62 | | 1.33 |  |  |  |  |  |  |  |  |
|  | | **General RL production model** | | | | | | | ***Pseudomonas* model** | | | | ***Burkholderia* model** | | | |
|  | | Minimum | Maximum | | Mean | | Std. deviation | | Minimum | Maximum | Mean | Std. deviation | Minimum | Maximum | Mean | Std. deviation |
| Concentration of Carbon, g/L | | 0.70 | 312.80 | | 19.15 | | 37.86 | | 0.70 | 194.00 | 19.23 | 35.23 | 5.91 | 312.80 | 26.37 | 60.01 |
| Molecular Volume of Carbon Source, Å^3^ | | 84.04 | 1081.00 | | 378.67 | | 373.41 | | 84.04 | 1081.00 | 389.78 | 372.31 | 87.12 | 976.50 | 382.18 | 417.75 |
| logP of carbon source | | -5.33 | 10.84 | | 2.12 | | 5.79 | | -5.33 | 10.84 | 2.37 | 5.79 | -2.60 | 10.72 | 2.39 | 5.86 |
| Concentration of Nitrogen, g/L | | 0.007 | 3.528 | | 1.154 | | 1.024 | | 0.007 | 3.528 | 1.061 | 0.985 | 0.231 | 2.799 | 1.676 | 1.117 |
| C:N ratio, w/w | | 0.4 | 1193.9 | | 33.3 | | 104.4 | | 0.4 | 1193.9 | 32.2 | 91.9 | 2.8 | 947.9 | 53.4 | 186.9 |
| pH of bacterial medium | | 6.0 | 10.0 | | 7.0 | | 0.4 | | 6.0 | 10.0 | 7.0 | 0.4 | 6.7 | 7.5 | 7.0 | 0.2 |
| Incubation Temperature, °C | | 20.0 | 55.0 | | 31.2 | | 4.3 | | 20.0 | 37.0 | 31.1 | 4.2 | 25.0 | 37.0 | 32.0 | 3.1 |
| Shaking Speed, rpm | | 100 | 600 | | 208 | | 62 | | 100 | 600 | 207 | 64 | 150 | 240 | 214 | 33 |
| Incubation Time, h | | 12 | 336 | | 117 | | 59 | | 12 | 336 | 117 | 55 | 24 | 264 | 129 | 76 |
| *log*P of RL | | 1.49 | 15.59 | | 5.07 | | 1.66 | | 1.49 | 15.59 | 4.80 | 1.36 | 2.64 | 9.00 | 7.66 | 1.31 |
